# Supplementary figures and images for: APOBEC3 Activity Promotes the Survival and Evolution of Drug-Tolerant Persister Cells during EGFR Inhibitor Resistance in Lung Cancer
Source: Cancer Res Commun. 2025 May 21;5(5):825–40. doi: 10.1158/2767-9764.CRC-24-0442 (PMC12093302; doi:10.1158/2767-9764.CRC-24-0442)

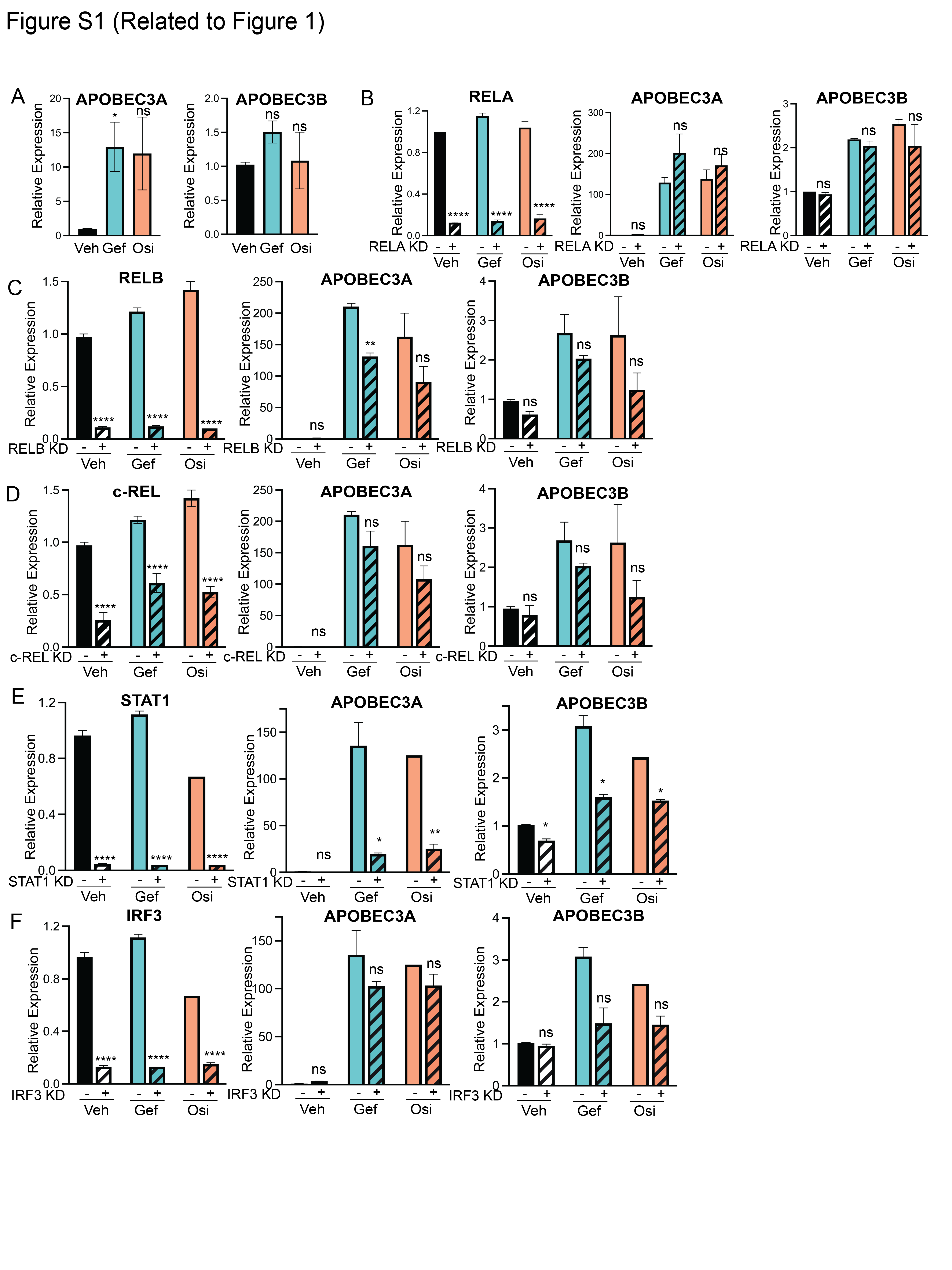

Supplement: Figure S1 — APOBEC expression is partially regulated by NFkappaB and STAT1. [file crc-24-0442_figure_s1_suppsf1.png]

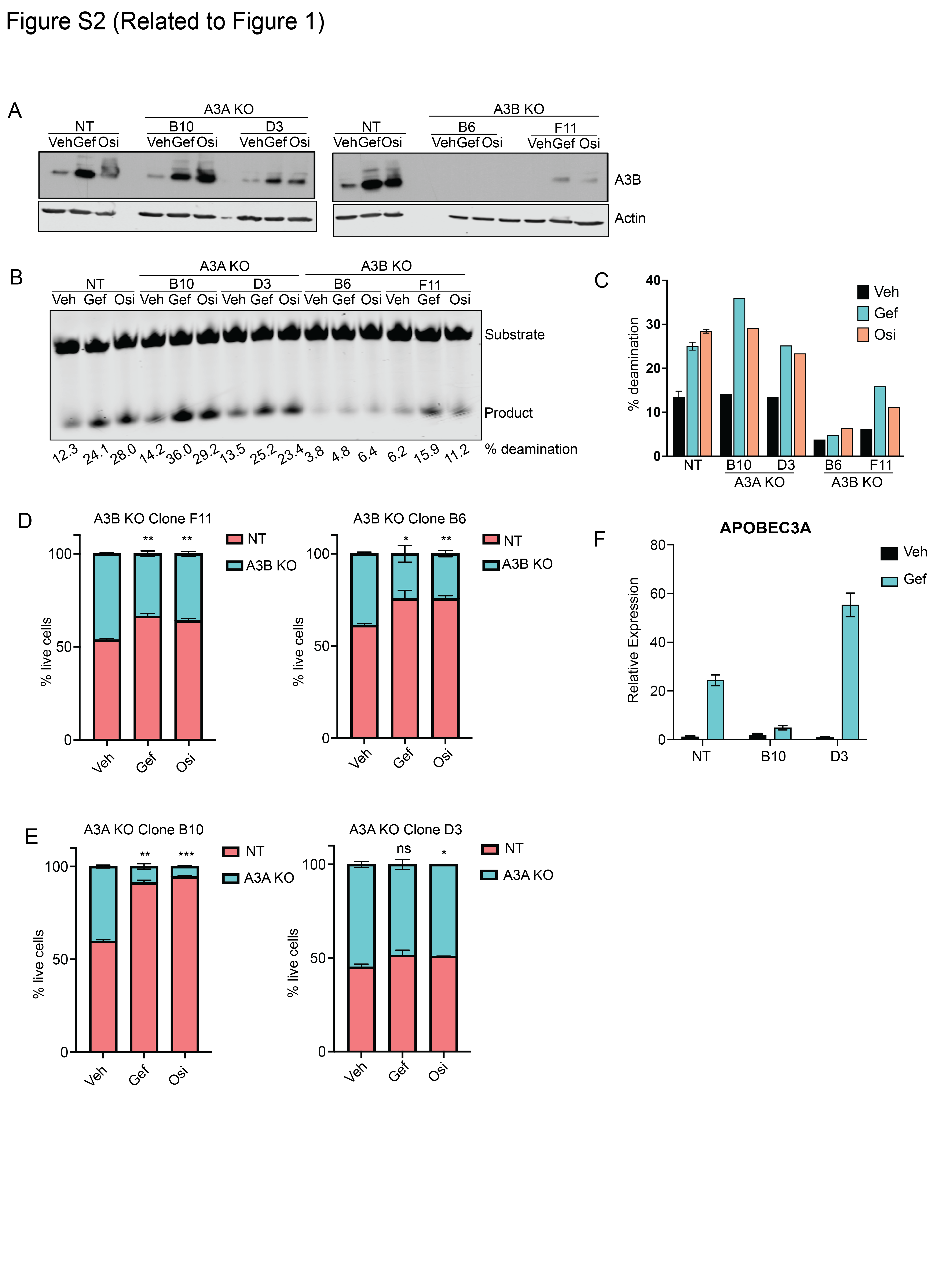

Supplement: Figure S2 — EGFR inhibitor-induced APOBEC activity is attenuated following single knockouts of A3A and A3B. [file crc-24-0442_figure_s2_suppsf2.png]

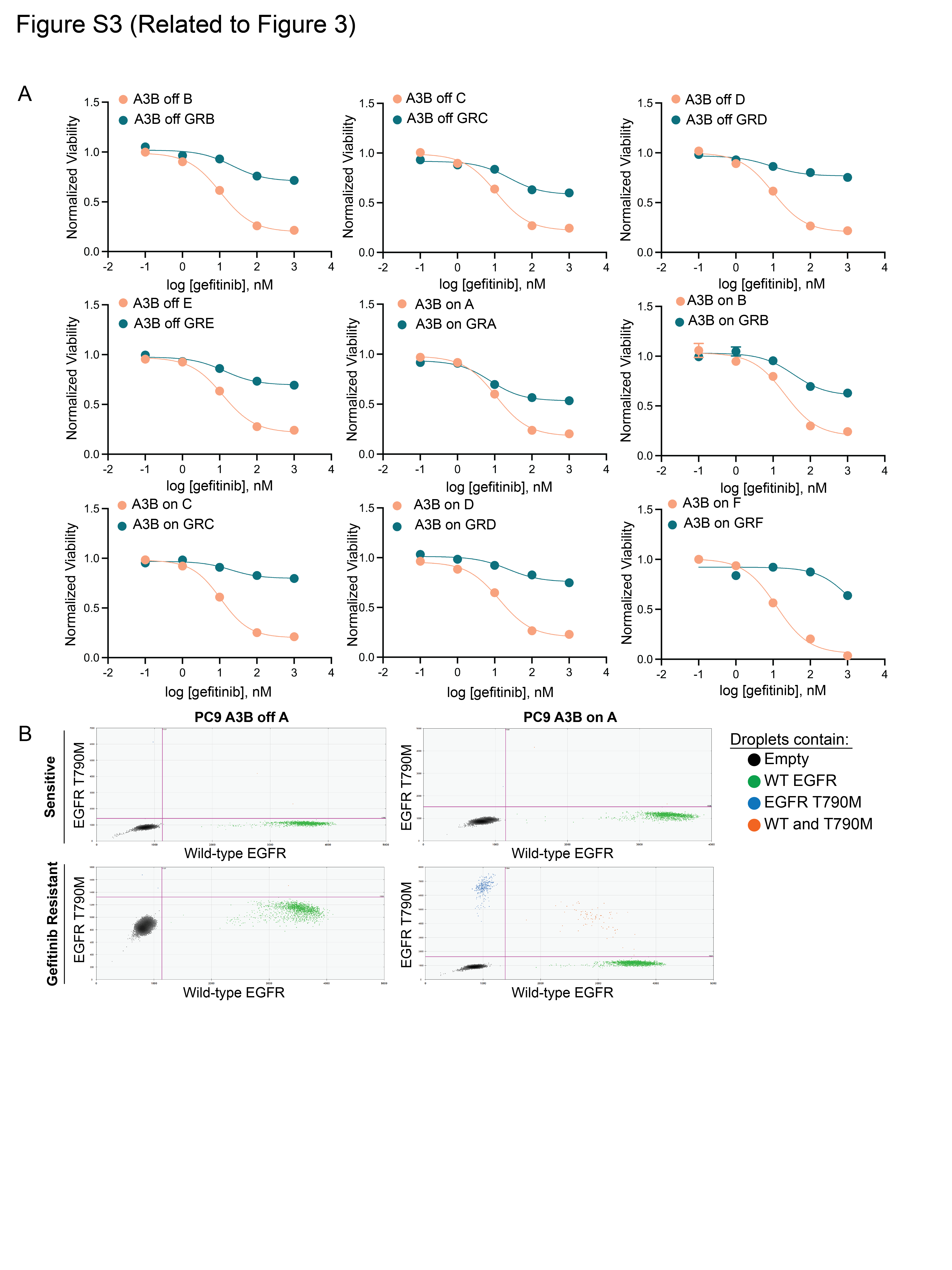

Supplement: Figure S3 — PC9 cells expressing APOBEC3B maintain high APOBEC activity following resistance to gefitinib. [file crc-24-0442_figure_s3_suppsf3.png]

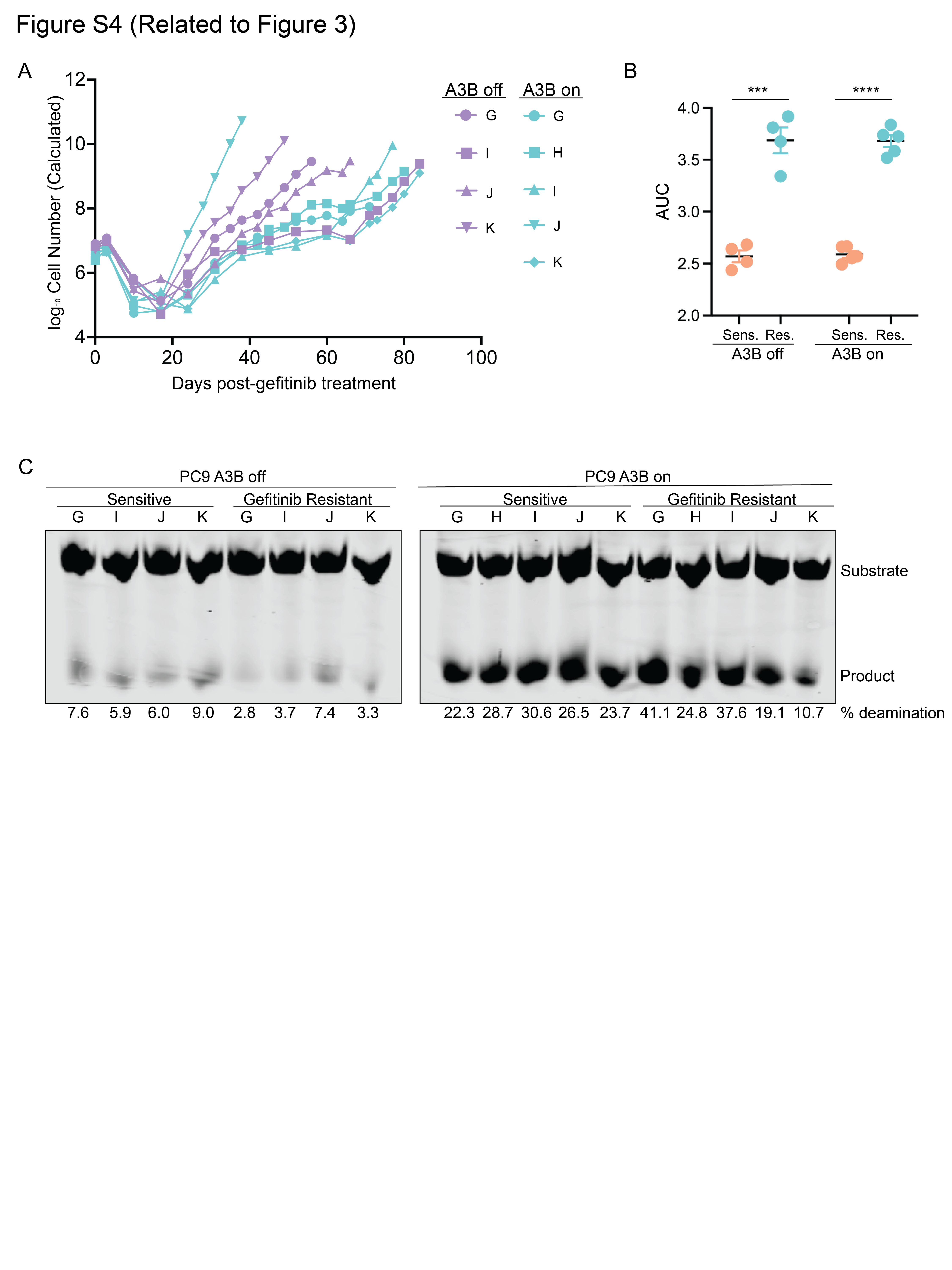

Supplement: Figure S4 — Developing resistance to gefitinib in A3B-off and A3B-on PC9 cells. [file crc-24-0442_figure_s4_suppsf4.png]

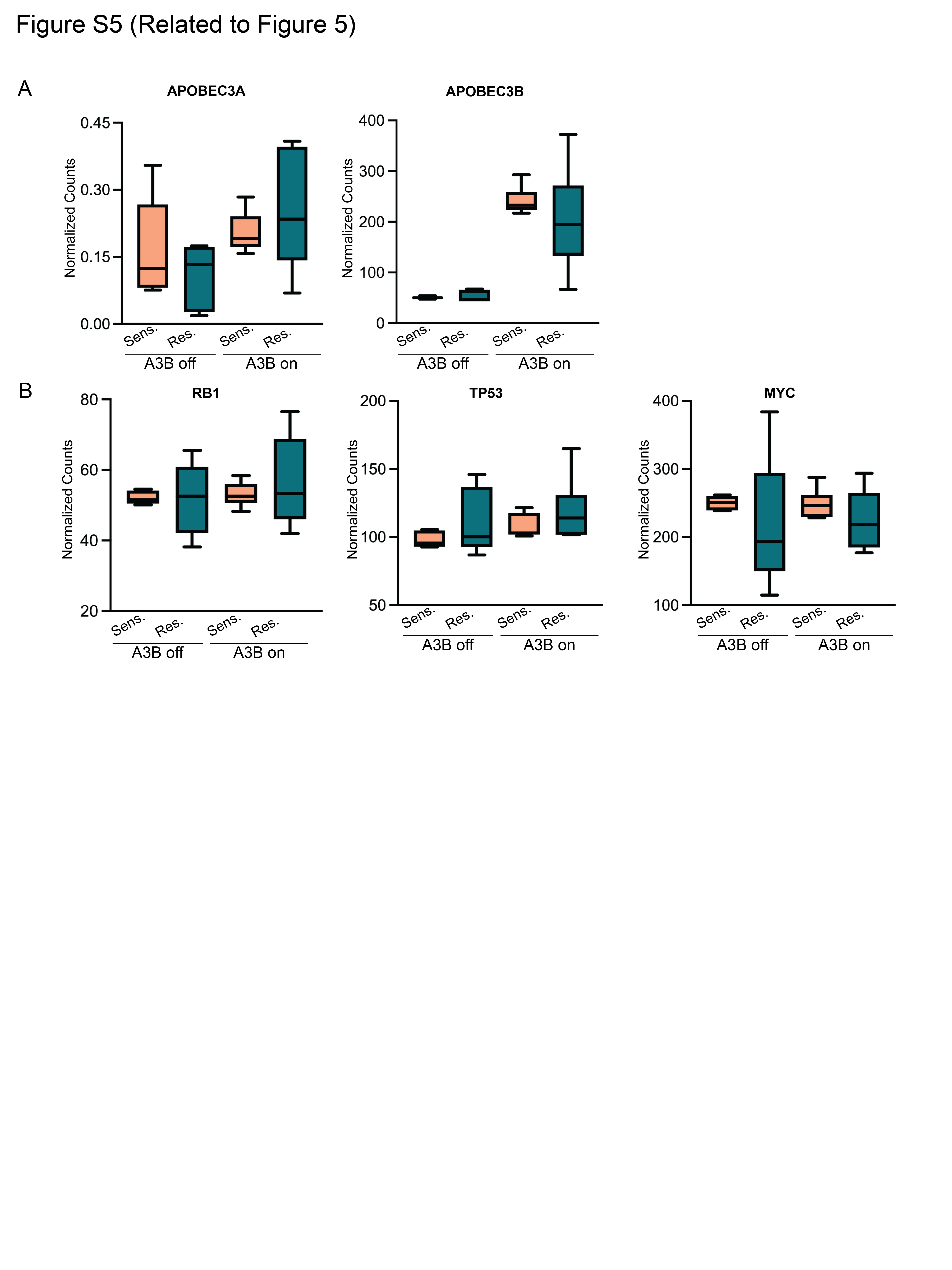

Supplement: Figure S5 — Gefitinib-resistant PC9 cells do not express markers of small-cell lung cancer transdifferentiation. [file crc-24-0442_figure_s5_suppsf5.png]

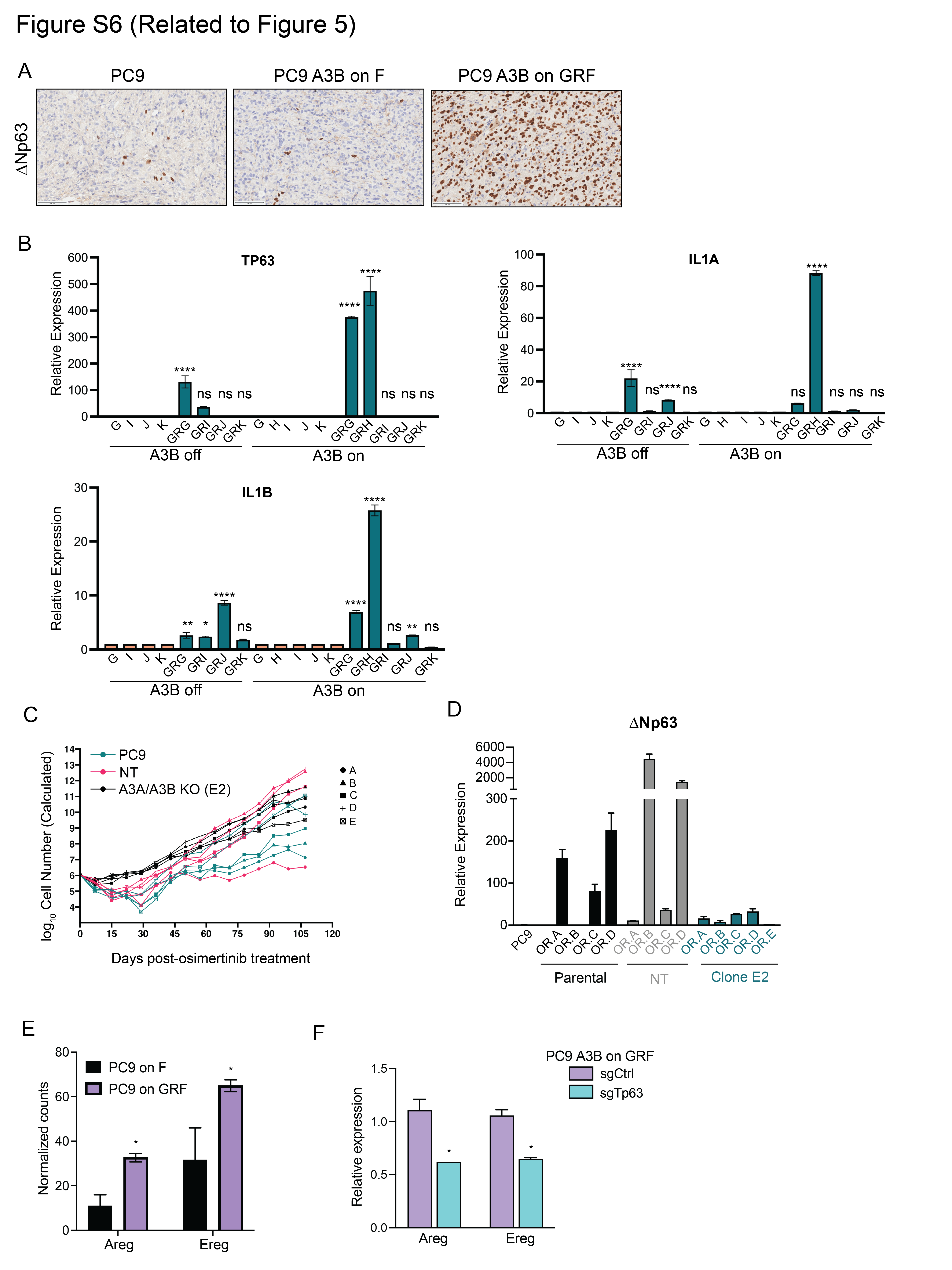

Supplement: Figure S6 — p63 and its target genes are highly expressed in A3B-on gefitinib-resistant PC9 cells. [file crc-24-0442_figure_s6_suppsf6.png]

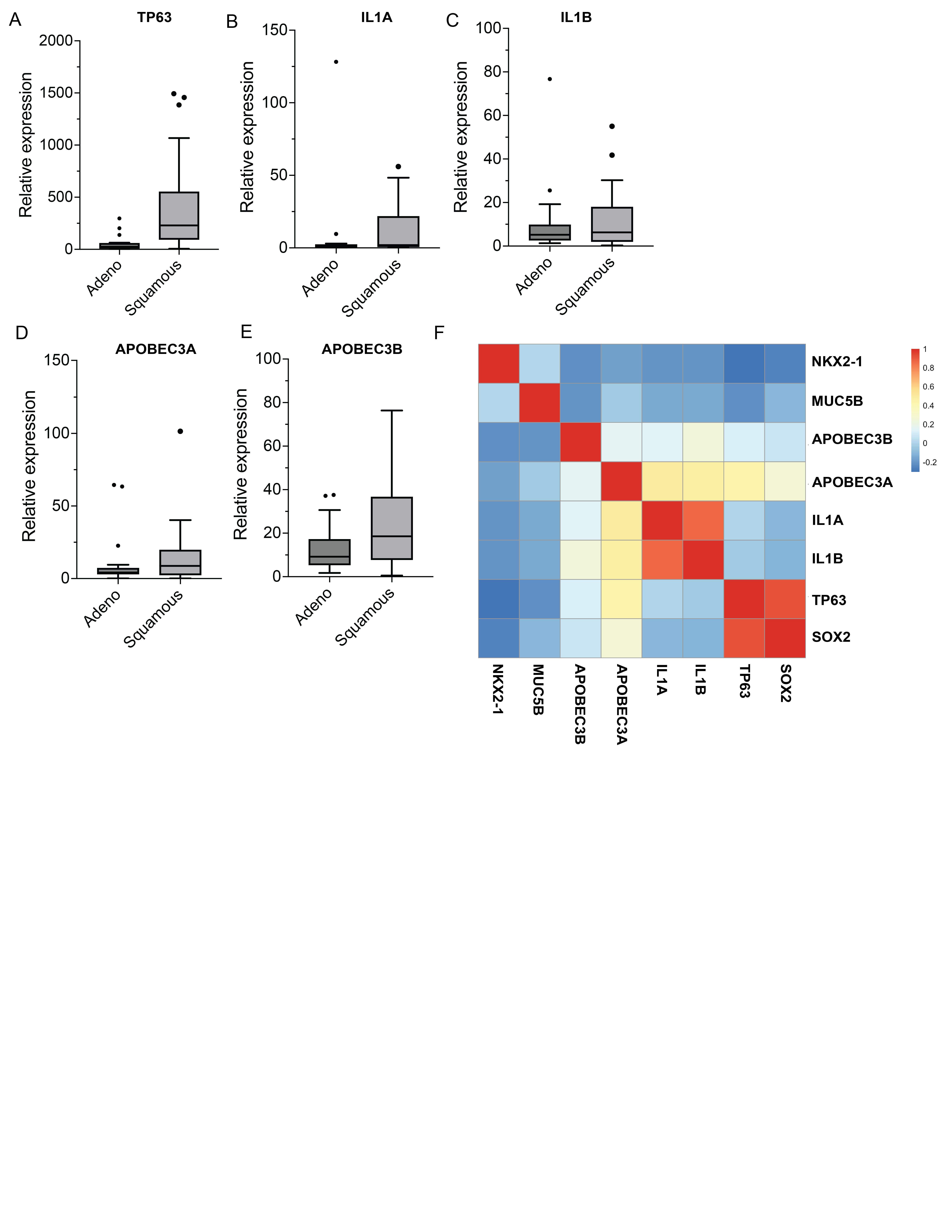

Supplement: Figure S7 — Expression of A3A/B and inflammatory genes in human lung tumors with adenocarcinoma or squamous histology. [file crc-24-0442_figure_s7_suppsf7.png]
