# Supplementary material for: APOBEC3 Activity Promotes the Survival and Evolution of Drug-Tolerant Persister Cells during EGFR Inhibitor Resistance in Lung Cancer
Source: Cancer Res Commun. 2025 May 21;5(5):825–40. doi: 10.1158/2767-9764.CRC-24-0442 (PMC12093302; doi:10.1158/2767-9764.CRC-24-0442)
Supplement: Table S1 — Sanger sequencing of individual bacterial colonies transformed with cloned PCR products of the region surrounding sgRNA recognition sites in A3A or A3B. [file crc-24-0442_table_s1_suppst1.docx]

**Supplementary Table S1.** Sanger sequencing of individual bacterial colonies transformed with cloned PCR products of the region surrounding sgRNA recognition sites in A3A or A3B.

Clone B10

APOBEC3A

| Sequence # | Insertion/Deletion | # bp | Sequence inserted/deleted | Location on mRNA | Predicted effect |
| --- | --- | --- | --- | --- | --- |
| 1 | Deletion | 17 | CCATCTTGACCGAGGTG | 212-228 | Frameshift |
| 2 | Insertion | 1 | A | 220-221 | Frameshift |
| 3 | Deletion | 17 | CCATCTTGACCGAGGTG | 212-228 | Frameshift |
| 4 | Deletion | 17 | CCATCTTGACCGAGGTG | 212-228 | Frameshift |
| 5 | Insertion | 1 | A | 220-221 | Frameshift |
| 6 | Insertion | 1 | A | 220-221 | Frameshift |
| 7 | Insertion | 1 | A | 220-221 | Frameshift |
| 8 | Insertion | 1 | A | 220-221 | Frameshift |
| 9 | Insertion | 3 | TTC | 175-176 | Frameshift |
|  | Deletion | 3 | CAG | 201-203 |  |
|  | Deletion | 14 | CTGGTCCATCTTGA | 220-233 |  |
| 10 | Deletion | 14 | CTGGTCCATCTTGA | 220-233 | Frameshift |
| 11 | Insertion | 1 | A | 220-221 | Frameshift |
| 12 | Deletion | 14 | CTGGTCCATCTTGA | 220-233 | Frameshift |
| 13 | Deletion | 14 | CTGGTCCATCTTGA | 220-233 | Frameshift |
| 14 | Insertion | 1 | A | 220-221 | Frameshift |
| 15 | Insertion | 1 | A | 220-221 | Frameshift |

­­

Clone D3

APOBEC3A

| Sequence # | Insertion/Deletion | # bp | Sequence inserted/deleted | Location on mRNA | Predicted effect |
| --- | --- | --- | --- | --- | --- |
| 1 | Deletion | 26 | TGCTGGTCCATCTTGACCGAGGTGCC | 210-235 | Frameshift |
| 2 | Deletion | 5 | CTTGA | 220-224 | Frameshift |
|  | Deletion | 5 | GGTCC | 227-231 | Frameshift |
| 3 | Deletion | 26 | TGCTGGTCCATCTTGACCGAGGTGCC | 210-235 | Frameshift |
| 4 | Insertion | 1 | A | 220-221 | Frameshift |
| 5 | Insertion | 6 | GTGCTG | 221-222 | No effect |
| 6 | Insertion | 1 | A | 220-221 | Frameshift |
| 7 | Insertion | 1 | A | 220-221 | Frameshift |
| 8 | Deletion | 26 | TGCTGGTCCATCTTGACCGAGGTGCC | 210-235 | Frameshift |
| 9 | Insertion | 6 | GTGCTG | 221-222 | No effect |
| 10 | Insertion | 1 | A | 220-221 | Frameshift |
| 11 | Insertion | 1 | A | 220-221 | Frameshift |
| 12 | Insertion | 1 | A | 220-221 | Frameshift |
| 13 | Insertion | 6 | GTGCTG | 221-222 | No effect |
| 14 | Deletion | 26 | TGCTGGTCCATCTTGACCGAGGTGCC | 210-235 | Frameshift |
| 15 | Insertion | 1 | A | 220-221 | Frameshift |

Clone B6

APOBEC3B

| Sequence # | Insertion/Deletion | # bp | Sequence inserted/deleted | Location on mRNA | Predicted effect |
| --- | --- | --- | --- | --- | --- |
| 1 | Deletion | 30 | ACCCTTTGGTCCTTCGACGGCGCCAGACCT | 721-750 | No effect |
| 2 | Deletion | 25 | CCTTTGGTCCTTCGACGGCGCCAGA | 723-747 | Frameshift |
| 3 | Sequencing failed |  |  |  |  |
| 4 | Deletion | 30 | ACCCTTTGGTCCTTCGACGGCGCCAGACCT | 721-750 | No effect |
| 5 | Deletion | 25 | CCTTTGGTCCTTCGACGGCGCCAGA | 723-747 | Frameshift |
| 6 | Sequencing failed |  |  |  |  |
| 7 | No mutations |  |  |  |  |
| 8 | Deletion | 30 | ACCCTTTGGTCCTTCGACGGCGCCAGACCT | 721-750 | No effect |
| 9 | Sequencing failed |  |  |  |  |
| 10 | Deletion | 30 | ACCCTTTGGTCCTTCGACGGCGCCAGACCT | 721-750 | No effect |
| 11 | Deletion | 25 | CCTTTGGTCCTTCGACGGCGCCAGA | 723-747 | Frameshift |
| 12 | Deletion | 30 | ACCCTTTGGTCCTTCGACGGCGCCAGACCT | 721-750 | No effect |
| 13 | Deletion | 25 | CCTTTGGTCCTTCGACGGCGCCAGA | 723-747 | Frameshift |
| 14 | Deletion | 25 | CCTTTGGTCCTTCGACGGCGCCAGA | 723-747 | Frameshift |
| 15 | Sequencing failed |  |  |  |  |

Clone F11

APOBEC3B

| Sequence # | Insertion/Deletion | # bp | Sequence inserted/deleted | Location on mRNA | Predicted effect |
| --- | --- | --- | --- | --- | --- |
| 1 | No mutations |  |  |  |  |
| 2 | No mutations |  |  |  |  |
| 3 | Sequencing failed |  |  |  |  |
| 4 | No mutations |  |  |  |  |
| 5 | Sequencing failed |  |  |  |  |
| 6 | Deletion | 47 | ATGACCCTTTGGTCCTTCGACGGCGCCAGACCTACTTGTGCTATGAG | 718-764 | Frameshift |
| 7 | No primer found |  |  |  |  |
| 8 | No mutations |  |  |  |  |
| 9 | Deletion | 47 | ATGACCCTTTGGTCCTTCGACGGCGCCAGACCTACTTGTGCTATGAG | 718-764 | Frameshift |
| 10 | Deletion | 47 | ATGACCCTTTGGTCCTTCGACGGCGCCAGACCTACTTGTGCTATGAG | 718-764 | Frameshift |
| 11 | Deletion | 47 | ATGACCCTTTGGTCCTTCGACGGCGCCAGACCTACTTGTGCTATGAG | 718-764 | Frameshift |
| 12 | Deletion | 47 | ATGACCCTTTGGTCCTTCGACGGCGCCAGACCTACTTGTGCTATGAG | 718-764 | Frameshift |
| 13 | Deletion | 32 | TGACCCTTTGGTCCTTCGACGGCGCCAGACCT | 719-750 | Frameshift |
| 14 | Deletion | 47 | CTCATAGCACAAGTAGGTCTGGCGCCGTCGAAGGACCAAAGGGTCAT | 718-764 | Frameshift |
| 15 | Deletion | 32 | TGACCCTTTGGTCCTTCGACGGCGCCAGACCT | 719-750 | Frameshift |

Clone E2

APOBEC3A

| Sequence # | Insertion/Deletion | # bp | Sequence inserted/deleted | Location on mRNA | Predicted effect |
| --- | --- | --- | --- | --- | --- |
| 1 | Deletion | 9 | CCATCTTGA | 220-228 | No effect |
| 2 | Deletion | 9 | CCATCTTGA | 220-228 | No effect |
| 3 | Deletion | 8 | CCGAGGTG | 212-219 | Frameshift |
| 4 | Insertion | 1 | A | 220-221 | Frameshift |
| 5 | Insertion | 1 | A | 220-221 | Frameshift |
| 6 | Deletion | 9 | CCATCTTGA | 220-228 | No effect |
| 7 | Deletion | 8 | CCGAGGTG | 212-219 | Frameshift |
| 8 | Insertion | 1 | A | 220-221 | Frameshift |
| 9 | Insertion | 1 | A | 220-221 | Frameshift |
| 10 | Insertion | 1 | A | 220-221 | Frameshift |
| 11 | Insertion | 4 | ATTG | 215-216 | Frameshift |
|  | Deletion | 3 | CCG | 217-219 |  |
| 12 | Insertion | 1 | A | 220-221 | Frameshift |
| 13 | Deletion | 8 | CCGAGGTG | 212-219 | Frameshift |
| 14 | Deletion | 4 | CGAG | 215-218 | Frameshift |
| 15 | Insertion | 1 | A | 220-221 | Frameshift |

Clone E2

APOBEC3B

| Sequence # | Insertion/Deletion | # bp | Sequence inserted/deleted | Location on mRNA | Predicted effect |
| --- | --- | --- | --- | --- | --- |
| 1 | No primer found |  |  |  |  |
| 2 | Deletion | 2 | CG | 735-736 | No effect |
|  | Insertion | 6 | TTATTT | 737-738 |  |
|  | Deletion | 2 | AG | 805-806 |  |
|  | Insertion | 2 | CT | 814-815 |  |
| 3 | Sequencing failed |  |  |  |  |
| 4 | Insertion | 4 | ATTA | 734-735 | Frameshift |
| 5 | Insertion | 1 | A | 734-735 | Frameshift |
| 6 | Sequencing failed |  |  |  |  |
| 7 | Insertion | 5 | ATTAA | 734-735 | Frameshift |
| 8 | Deletion | 2 | CG | 735-736 | Frameshift |
|  | Insertion | 6 | TTAATT | 737-738 |  |
| 9 | Deletion | 22 | TCGACGGCGCCAGACCTACTTG | 734-755 | Frameshift |
| 10 | Insertion | 7 | ATTATTA | 734-735 | Frameshift |
|  | Deletion | 5 | CGGCG | 738-742 |  |
| 11 | Deletion | 22 | TCGACGGCGCCAGACCTACTTG | 734-755 | Frameshift |
| 12 | Insertion | 11 | TATTATTATTA | 733-734 | Frameshift |
|  | Deletion | 7 | GACGGCG | 736-742 |  |
|  | Deletion | 2 | AG | 745-746 |  |
| 13 | Deletion | 22 | TCGACGGCGCCAGACCTACTTG | 734-755 | Frameshift |
| 14 | Insertion | 4 | ATTA | 734-735 | Frameshift |
| 15 | Deletion | 22 | TCGACGGCGCCAGACCTACTTG | 734-755 | Frameshift |

Clone G10

APOBEC3A

| Sequence # | Insertion/Deletion | # bp | Sequence inserted/deleted | Location on mRNA | Predicted effect |
| --- | --- | --- | --- | --- | --- |
| 1 | Deletion | 13 | TGCTGGTCCATCT | 223-235 | Frameshift |
| 2 | Insertion | 1 | A | 720-721 | Frameshift |
| 3 | Deletion | 6 | TGACCG | 217-222 | No effect |
| 4 | Deletion | 13 | TGCTGGTCCATCT | 223-235 | Frameshift |
| 5 | Deletion | 13 | TGCTGGTCCATCT | 223-235 | Frameshift |
| 6 | Insertion | 1 | A | 220-221 | Frameshift |
| 7 | Insertion | 1 | A | 220-221 | Frameshift |
| 8 | Insertion | 1 | A | 220-221 | Frameshift |
| 9 | Insertion | 1 | A | 220-221 | Frameshift |
| 10 | Insertion | 1 | A | 220-221 | Frameshift |
| 11 | Insertion | 1 | A | 220-221 | Frameshift |
| 12 | Sequencing failed |  |  |  |  |
| 13 | Insertion | 1 | A | 220-221 | Frameshift |
| 14 | Deletion | 13 | TGCTGGTCCATCT | 223-235 | Frameshift |
| 15 | Insertion | 1 | A | 220-221 | Frameshift |

Clone G10

APOBEC3B

| Sequence # | Insertion/Deletion | # bp | Sequence inserted/deleted | Location on mRNA | Predicted effect |
| --- | --- | --- | --- | --- | --- |
| 1 | Sequencing failed |  |  |  |  |
| 2 | Deletion | 26 | GACCCTTTGGTCCTTCGACGGCGCCA | 720-745 | Frameshift |
| 3 | No mutations |  |  |  | No effect |
| 4 | Deletion | 26 | GACCCTTTGGTCCTTCGACGGCGCCA | 720-745 | Frameshift |
| 5 | Substitution | 1 | A->T | 763 | Substitution |
| 6 | Deletion | 26 | GACCCTTTGGTCCTTCGACGGCGCCA | 720-745 | Frameshift |
| 7 | No mutations |  |  |  |  |
| 8 | Sequencing failed |  |  |  |  |
| 9 | No mutations |  |  |  |  |
| 10 | No mutations |  |  |  |  |
| 11 | No mutations |  |  |  |  |
| 12 | Deletion | 26 | GACCCTTTGGTCCTTCGACGGCGCCA | 720-745 | Frameshift |
| 13 | No mutations |  |  |  |  |
| 14 | No mutations |  |  |  |  |
| 15 | No mutations |  |  |  |  |
